# Quantifying human mixing patterns in Chinese provinces outside Hubei after the 2020 lockdown was lifted

**DOI:** 10.1186/s12879-022-07455-7

**Published:** 2022-05-21

**Authors:** Yining Zhao, Samantha O’Dell, Xiaohan Yang, Jingyi Liao, Kexin Yang, Laura Fumanelli, Tao Zhou, Jiancheng Lv, Marco Ajelli, Quan-Hui Liu

**Affiliations:** 1 College of Computer Science, Sichuan University, Chengdu, China; 2 Laboratory for Computational Epidemiology and Public Health, Department of Epidemiology and Biostatistics, Indiana University School of Public Health, Bloomington, IN USA; 3 Institute for Applied Computational Science, Harvard University, Cambridge, MA USA; 4 Shenzhen International Graduate School, Tsinghua University, Shenzhen, China; 5 Big Data Research Center, University of Electronic Science and Technology of China, Chengdu, China

**Keywords:** Contact patterns, Human behavior, COVID-19, Age, Disease burden, Mathematical modeling

## Abstract

**Background:**

Contact patterns play a key role in the spread of respiratory infectious diseases in human populations. During the COVID-19 pandemic, the regular contact patterns of the population have been disrupted due to social distancing both imposed by the authorities and individual choices. Many studies have focused on age-mixing patterns before the COVID-19 pandemic, but they provide very little information about the mixing patterns in the COVID-19 era. In this study, we aim at quantifying human heterogeneous mixing patterns immediately after lockdowns implemented to contain COVID-19 spread in China were lifted. We also provide an illustrative example of how the collected mixing patterns can be used in a simulation study of SARS-CoV-2 transmission.

**Methods and results:**

In this work, a contact survey was conducted in Chinese provinces outside Hubei in March 2020, right after lockdowns were lifted. We then leveraged the estimated mixing patterns to calibrate a mathematical model of SARS-CoV-2 transmission. Study participants reported 2.3 contacts per day (IQR: 1.0–3.0) and the mean per-contact duration was 7.0 h (IQR: 1.0–10.0). No significant differences in average contact number and contact duration were observed between provinces, the number of recorded contacts did not show a clear trend by age, and most of the recorded contacts occurred with family members (about 78%). The simulation study highlights the importance of considering age-specific contact patterns to estimate the COVID-19 burden.

**Conclusions:**

Our findings suggest that, despite lockdowns were no longer in place at the time of the survey, people were still heavily limiting their contacts as compared to the pre-pandemic situation.

**Supplementary Information:**

The online version contains supplementary material available at 10.1186/s12879-022-07455-7.

## Introduction

The COVID-19 pandemic has caused countries around the globe to adopt unprecedented measures to combat the spread of the disease. China has adopted lockdowns and social distancing strategies to contain the spread of SARS-CoV-2 [1]. As a result, provinces outside Hubei were able to quickly contain the spread of infection [2]. Since February 24, 2020, lockdowns were lifted in all provinces except for Hubei and Beijing [3]; however, many individuals were still hesitant to resume normal activities. This behavioral change is a significant indicator of how mixing patterns have changed throughout the population during the pandemic.

Before the COVID-19 pandemic, many studies have focused on age-mixing patterns to measure the features of contacts between individuals in different age groups and to understand the spread of infectious diseases [4–8]. Those patterns, however, are representative of a pre-pandemic situation and have little in common with life during this pandemic [4]. As shown by human mobility data (e.g., Google, Apple, Yandex, Weibo [9–12]), activity levels have dramatically changed over the course of the pandemic, compared to pre-pandemic conditions. However, this data does not provide any estimate of the heterogeneous mixing patterns by age groups or other descriptive measures needed to fully understand COVID-19 epidemiology [13, 14]. As a result, only a handful of studies have analyzed to what extent mixing patterns by age have changed during the pandemic as compared to pre-pandemic conditions [15–17].

The aim of this study is to estimate age-mixing patterns relevant to the spread of SARS-CoV-2 in Chinese provinces outside Hubei over the period of March 3–23, 2020 through survey-based contact diaries and to present an application of the collected contact patterns data to investigate SARS-CoV-2 transmission. During this period, lockdowns had just lifted, but social distancing was still in place. Individuals who participated in the survey were asked to record all persons they were in contact with over a 24-h period, with contact being defined as exchanging more than three words in physical presence or having a physical contact. Participants were then asked to define each contact by age, sex, employment status, relationship to the contact, the social setting in which the contact took place, and the duration of the interaction. Next, we leveraged a mathematical model calibrated using the estimated contact patterns to provide an illustrative example of the use of age-mixing patterns to provide insights on their role in SARS-Cov-2 transmission as compared to a model assuming a homogeneous mixing of the population.

## Methods

### Survey on contact patterns

To estimate age-mixing patterns, we performed a contact survey by Wenjuanxing, a professional online survey platform widely adopted in China [18]. Participants filled in the survey between March 3, 2020 and March 23, 2020. Lockdowns (formally, Level 1 Public Health Emergency Response) were imposed in all Chinese provinces between January 23 and January 25, 2020 [3]. As of February 24, 2020, in all provinces except Hubei and Beijing, the transport network was restored, and the State Council advised local authorities and governments to ensure a return to normal [3]. However, although lockdowns were lifted, other non-pharmacological interventions (NPIs) were still in place. Those interventions included school closure, promotion of remote working, temperature taking to access public places, wearing masks in public, prohibiting mass gatherings, and scanning HealthQR codes. Only provinces with at least 10 participants were included in the analysis. Hubei Province was not included in this analysis, as Wuhan (its largest city) remained in lockdown during this time and its epidemiological situation was highly different as compared to the rest of the country.

When the survey was first given to the participants, the background and purpose of the survey were explained in detail. Confidentiality was also guaranteed to each participant for all personal information collected. Additionally, participants signed confirmation of authenticity to ensure that all information they provided was correct. They also agreed to have their IP addresses automatically taken from the platform to record the Chinese Province where they were based at the time of the survey.

The survey consisted of two areas of questioning: (1) demographic information; and (2) social contact information. The demographic information questions regarded participants’ age, sex, province of residence, as well as the age and sex of all members of the participants’ households.

The contact diary was then assigned to the participants to record all individuals they came into contact with in a 24-h time period. A contact was defined as either: (1) a two-way conversation that involved at least 3 words in the physical presence of another person (conventional contact), or (2) a direct physical contact (e.g., a handshake, hug, kiss) [4]. As a result, only individuals that the participants interacted with that met these conditions were included in the contact diaries. For each contact, the participants recorded the age (or age range if age was unknown); their relationship with the contact (household member, other relative, classmate/colleague, other schoolmate, other); where the contact took place (house, work, school, leisure, transport, other); and contact duration. Contact duration was defined as the amount of time spent in the same room/environment with a contacted individual. If a participant had a contact with the same individual several times during the day, they were instructed to record the contact only once and cumulated the total time spent with that person in any location.

### Statistical analysis

To quantify the average number of contacts and contact duration for each age group and to construct contact matrices by age, we performed bootstrap sampling [19] with replacement of survey participants weighted by the age distribution for the 12 provinces included in our analysis as reported in the 2010 Chinese census [20]. Every cell of each matrix represents the average of 100 bootstrapped realizations. The age distributions of study participants, the 12 analyzed provinces and the bootstrapped population are reported in Additional file 1: Fig. S1.

We estimated the mean number of contacts and their mean duration from the survey, both irrespective of age and for each age group. Contact patterns between individuals in the same age groups are shown as well given their relevance in defining the assortativity of the contact matrix and, ultimately, the value of the reproduction number of an infection in that population [21, 22]. We then scaled to the actual population of China: the mean number of contacts weighted by the age distribution for the 12 provinces included in our analysis as reported in the 2010 Chinese census gives the population-adjusted mean number of contacts. By an analogous procedure we obtained the population-adjusted mean duration of contacts. Mann Whitney with the corresponding independent sample t-test was used to determine whether differences (e.g., by province) in the number of contacts and duration were statistically significant.

### Modeling SARS-CoV-2 transmission

We developed a mathematical model of the infection transmission process as an illustrative example of how the measured contact patterns by age can be used to provide estimates of SARS-CoV-2 transmission dynamics and COVID-19 burden. Therefore, an SIR model is developed. Briefly, the population is divided into three compartments: susceptible (S), representing individuals who can acquire the infection; infectious (I), representing individuals who are infected and able to transmit the infection; and removed (R), representing individuals who are immune to the infection. Each compartment is divided into 18 5-year age groups (0–4, 5–9,…, 80–84, 85+). Susceptible individuals are exposed to an age-specific force of infection that depends on the number of infectious individuals of a given age, the matrix of contacts regulating the intensity (i.e., number and duration) of contacts with individuals of a given age per unit of time, and the transmission rate per contact. Infectious individuals move to the removed compartment according to a recovery rate. This process is regulated by the following system of ordinary differential equations:

$${\dot{S}}_{i}=-{\mathrm{S}}_{\mathrm{i}}\upbeta {\sum }_{\mathrm{j}=1}^{\mathrm{n}}{M}_{g\left(i\right)g\left(j\right)}{T}_{g\left(i\right)g\left(j\right)}\frac{{\sum }_{k=1,\dots ,m}{I}_{k}{\updelta }_{g\left(k\right)g\left(j\right)}}{{\sum }_{k=1,\dots ,m}{N}_{k}{\updelta }_{g\left(k\right)g\left(j\right)}},$$

$${\dot{I}}_{i}{=\mathrm{S}}_{\mathrm{i}}\upbeta {\sum }_{\mathrm{j}=1}^{\mathrm{n}}{M}_{g\left(i\right)g(j)}{T}_{g\left(i\right)g(j)}\frac{{\sum }_{k=1,\dots ,m}{I}_{k}{\updelta }_{g\left(k\right)g\left(j\right)}}{{\sum }_{k=1,\dots ,m}{N}_{k}{\updelta }_{g\left(k\right)g\left(j\right)}}-\gamma {I}_{i},$$

$${\dot{R}}_{i}=\gamma {I}_{i},$$

where, ${\mathrm{S}}_{\mathrm{i}}$ represents the number of susceptible individuals in age group i; ${\mathrm{I}}_{\mathrm{i}}$ represents the number of infectious individuals in age group i; ${\mathrm{R}}_{\mathrm{i}}$ represents the number of removed individuals in age group i; ${\mathrm{N}}_{\mathrm{i}}$ represents the total number of individuals in age group i (i.e., ${\mathrm{N}}_{\mathrm{i}}={\mathrm{S}}_{\mathrm{i}}+{\mathrm{I}}_{\mathrm{i}}+{\mathrm{R}}_{\mathrm{i}}$); $g( )$ is a function used to map the n = 18 age groups used for the population to the *m* = 6 age groups used in the contact matrix. Therein,

$$g(k)=\left\{\begin{array}{l} 1, \text { if } k \leq 4(\text { i.e., age } \in[0,19]), \\ 2, \text { if } k=5 (\text { i.e., age } \in[20,24]), \\ 3, \text { if } k=6 (\text { i.e., age } \in[25,29]), \\ 4, \text { if } k=7 (\text { i.e., age } \in[30,34]), \\ 5, \text { if } k=8 (\text { i.e., age } \in[35,39]), \\ 6, \text { if } k \geq 9 (\text { i.e., age } \in[40,100]). \end{array}\right.$$

${\mathrm{M}}_{\mathrm{g}(\mathrm{i})\mathrm{g}(\mathrm{j})}$ is the mean number of contacts that an individual in age group g(i) has with individuals in age group g(j); ${\mathrm{T}}_{\mathrm{g}\left(\mathrm{i}\right)\mathrm{g}(\mathrm{j})}$ is the mean duration of a contact that an individual in age group g(i) has with individuals in age group g(j); ${\updelta }_{g\left(k\right)g\left(j\right)}$ is the Kronecker delta function (i.e., it is equal to 1 if g(k) = g(j); 0 otherwise); $\upbeta$ is the per-contact transmission rate per hour; $\upgamma$ is the recovery rate, which corresponds to the inverse of the generation time in an SIR model [23, 24], and it is set to 5.1 days [2].

A key parameter regulating the spread of the infection is the basic reproduction number, ${\mathrm{R}}_{0}$, representing the average number of infections generated by a typical primary infector in a fully susceptible population over the whole duration of their infectious period [22]. We used the next-generation matrix approach [21] to calculate the per-contact transmission per hour (β) given a specified value of ${\mathrm{R}}_{0}$. Namely, β = (${\mathrm{R}}_{0}$ γ)/ρ(Q), where ρ(Q) is the dominant eigenvalue of the contact matrix.

Model simulations for the baseline are initialized with one infected individual, whose age group is randomly sampled proportional to the size of the age group. Simulations are then run until 1000 cumulative infections are reached. The reproduction number was set to 1.3. The rationale for these choices is that we want to represent a situation similar to that of the Chinese provinces outside Hubei in the period right antecedent to our contact survey, where strict interventions were quickly enacted after a COVID-19 outbreak was detected [2]. Moreover, a similar approach has been used by Chinese authorities for the entire duration of the COVID-19 pandemic. As such, it would be unlikely that contact patterns would remain unaltered for a prolonged period, extending beyond the detection of the first 1,000 infections.

We conducted three sensitivity analyses where we varied the number of initial seeds (1; 5; 20), the reproduction number (1.3; 2.0), and the cumulative number of infections to interrupt the simulation (1000; 500; 20,000).

Along with the infection transmission model, we developed a disease burden model. This model takes as input the number of infected individuals by age estimated by the transmission model and applies age-specific risks to estimate the distributions by age of some major indicators of disease burden: symptomatic infections, hospital admissions, and deaths. The values of these age-specific risks are taken from the literature [25, 26] and reported in Additional file 1: Table S1.

To show to what extent age mixing patterns affect SARS-CoV-2 transmission and COVID-19 burden, we run the model using three assumptions on the mixing patterns of the population: homogeneous mixing (contacts are the same for any individual of the population, irrespective of their age), contact matrix (contacts are based on the recorded number of contacts by age only), and contact and duration matrix (contacts are based on the recorded number of contacts by age and their duration). Parameters used in model simulations are reported in Additional file 1: Table S2.

## Results

### Description of the sample

In total, we collected 748 diaries; 394 participants were excluded from the study. Of these excluded participants, 311 did not have complete contact or residential information, 14 did not reside in Mainland China outside Hubei, and 1 responded to the survey outside of the study period. In the remaining 422 clean diaries, 200 diaries are from Sichuan. Outside Sichuan, only participants from the 12 provinces with at least 10 diaries were kept, which brings the total number diaries analyzed in this study to 354 (Additional file 1: Table S3). No significant difference in both contact numbers and contact duration with Sichuan were found for these provinces (Mann Whitney test, all p-values > 0.01), except for Shanxi. Shanxi Province exhibited an average contact duration of 9.7 h as compared to an overall average of 7.0 h.

### Number of contacts and duration

A total of 828 contacts were analyzed in this study. The mean number of daily contacts was 2.3 (median: 2; interquartile range, IQR: 1.0–3.0) (Table 1). The overall mean contact duration was 7.0 h (IQR: 1.0–10.0).

**Table 1:** Number and duration of recorded contacts per participant

Characteristics	Number of participants	Contact number	Contact duration
		Mean (median; IQR)	Mean (median; IQR)
Overall	354	2.3 (2.0; 1.0, 3.0)	7.0 (5.0; 1.0, 10.0)
Age of participant			
0–19	53	2.3 (2.0; 2.0, 3.0)	7.5 (7.1; 1.2, 12.0)
20–24	141	2.4 (2.0; 1.0, 3.0)	7.0 (6.8; 1.8, 10.0)
25–29	79	2.0 (2.0; 1.0, 3.0)	7.9 (6.9; 0.8, 10.0)
30–34	36	2.8 (2.0; 1.0, 4.0)	5.9 (6.2; 0.4, 11.0)
35–39	18	3.1 (2.0; 2.0, 4.0)	6.6 (6.6; 2.2, 10.0)
40 +	27	2.0 (2.0; 1.0, 3.0)	5.4 (4.9; 0.3, 7.8)
Sex of participant^a^			
Male	189	2.4 (2.0; 1.0, 3.0)	6.4 (4.6; 1.0, 10.0)
Female	164	2.3 (2.0; 1.0, 3.0)	6.9 (5.0; 1.4, 10.0)
Household size			
1	20	1.7 (1.0; 0.0, 2.0)	2.9 (0.4; 0.0, 6.0)
2	108	1.6 (1.0; 1.0, 2.0)	6.1 (3.0; 0.5, 10.0)
3	121	2.2 (2.0; 2.0, 2.0)	6.9 (5.5; 1.8, 10.0)
4	76	2.9 (3.0; 2.0, 3.0)	7.4 (6.1; 2.0, 10.6)
5	19	4.1 (4.0; 4.0, 4.0)	7.5 (6.6; 3.0, 9.6)
6 +	10	6.6 (5.5; 4.0, 11.0)	9.0 (4.1; 2.3, 20.0)
Provinces			
Sichuan Chongqing	200 20	2.3 (2.0; 1.0, 3.0) 2.8 (2.0; 1.0, 4.0)	6.5 (4.6; 0.8, 10.0) 7.6 (6.1; 2.5, 12.0)
Shandong	19	2.1 (2.0; 1.0, 2.0)	7.0 (3.0; 0.5, 11.7)
Hebei	17	2.1 (2.0; 2.0, 2.0)	7.4 (6.1; 2.0, 10.0)
Henan	16	2.7 (2.0; 2.0, 3.0)	5.1 (2.3; 0.8, 11.0)
Zhejiang	14	2.4 (2.0; 2.0, 3.0)	5.8 (4.8; 0.9, 12.0)
Yunnan	13	3.1 (2.0; 1.0, 3.0)	5.5 (6.8; 1.5, 9.6)
Fujian	13	2.2 (2.0; 2.0, 2.0)	6.3 (5.0; 1.0, 8.2)
Hunan	12	1.9 (2.0; 1.0, 3.0)	7.0 (5.5; 1.0,11.3)
Guangdong	10	2.2 (2.0; 1.0, 3.0)	7.2 (4.0; 2.0,10.0)
Jiangxi	10	3.0 (2.5; 2.0, 4.0)	6.6 (3.8; 2.2,10.0)
Shanxi	10	2.8 (2.0; 2.0, 3.0)	9.7 (8.8; 5.0,12.5)

^a^Note that one participant refused to fill in their gender

### Contact patterns by gender, age, relationship, and location

**Fig. 1:**
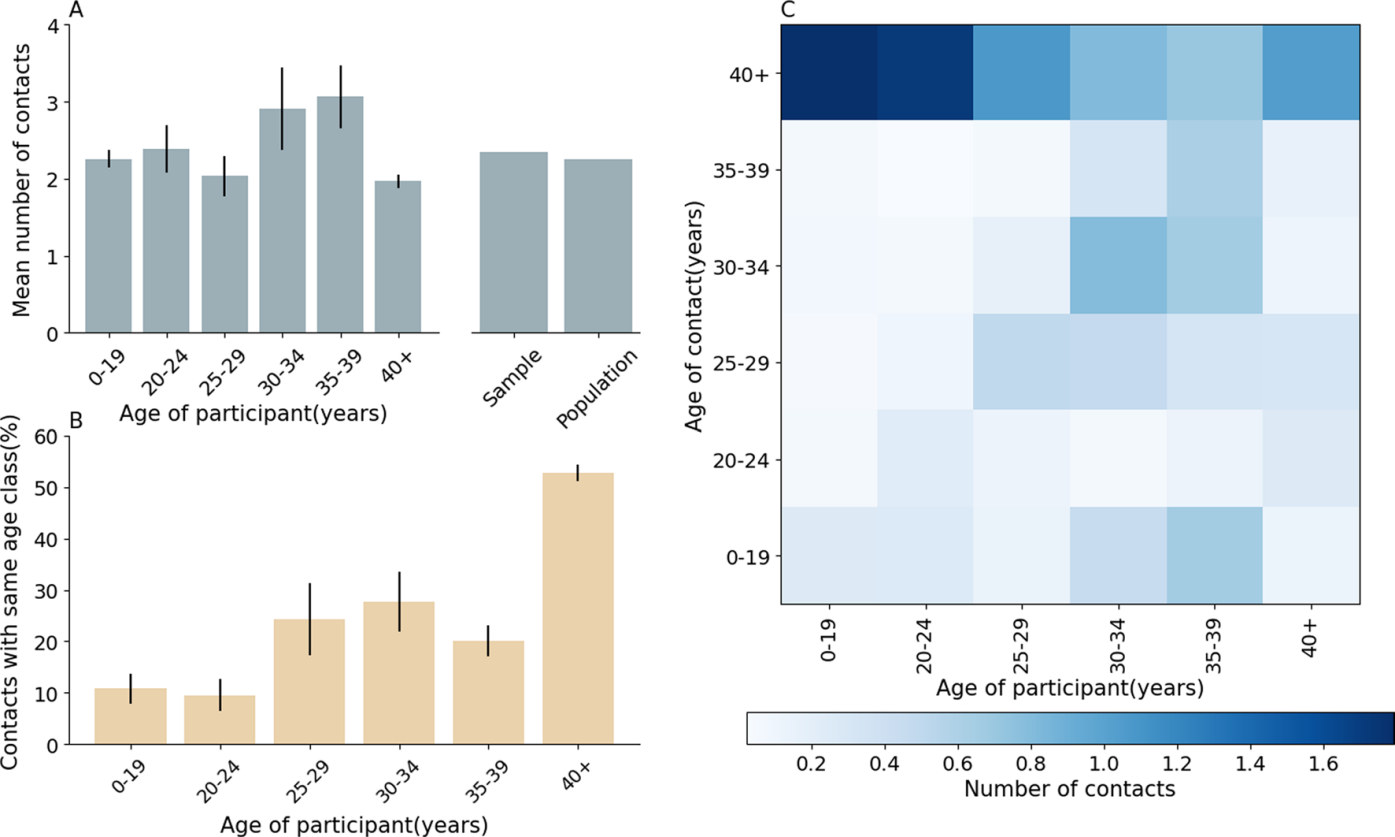
Daily number of contacts and contacts by age. **A** Mean number of contacts by age group of study participants. The standard error in each age group is obtained from bootstrap sampling according to the age distribution for the 12 provinces included in our analysis as reported in the 2010 Chinese census. The two bars on the right show the mean number of contacts irrespective of the age of study participant in the sample and by adjusting for the age structure of the 12 provinces included in our analysis. **B** Fraction of contacts with same age class as participants. **C** Overall contact matrix by age group

Individuals with different genders demonstrated no apparent distinctions in their contact numbers and duration, resulting in 2.3–2.4 contacts and 6.4–6.9 h respectively (Mann Whitney test, p-values > 0.01). The average number of contacts increased as the household size became larger, rising from 1.7 with a household size of one to 6.6 for a household size of six or more. The average contact duration also exhibited the same pattern, increasing from 2.9 for a household of one to 9.0 for households with six or more members. The mean number of contacts by age shows only slight differences in individuals aged from 30–34 and 35–39. Individuals in the 30–34 age group and individuals in the 35–39 age group exhibited daily mean contacts as 2.8 and 3.1 respectively, which is slightly higher than the daily mean contacts for individuals in the other age groups (Table 1). Since the numbers of participants in the six age groups were unbalanced, we performed bootstrap sampling with replacement of survey participants weighted by the age distribution for the 12 provinces included in our analysis as reported in the 2010 Chinese census [20]. We then recalculated the population-adjusted mean number of contacts in this fashion. It can be discerned that there were no obvious differences obtained in the distribution of contact numbers after bootstrap sampling (Fig. 1A).

In Fig. 1B, individuals in age groups 0–19 and 20–24 have the lowest numbers of same-age contacts, while individuals in the 40 + age group had the highest number. This finding was possibly explained by the fact that these age groups are almost students in the population and their school semester was postponed during the time of the study.

Mixing patterns by age can be summarized in a contact matrix, whose elements represent the mean number of contacts that an individual in a given age group has with individuals in other age groups (Fig. 1C). The resulting contact matrix by age is characterized by the presence of a main diagonal representing contacts with individuals in the same age group. Contacts between school age individuals are lower than 1 per day on average; in fact, most schools were still closed in the analyzed time frame.

**Fig. 2:**
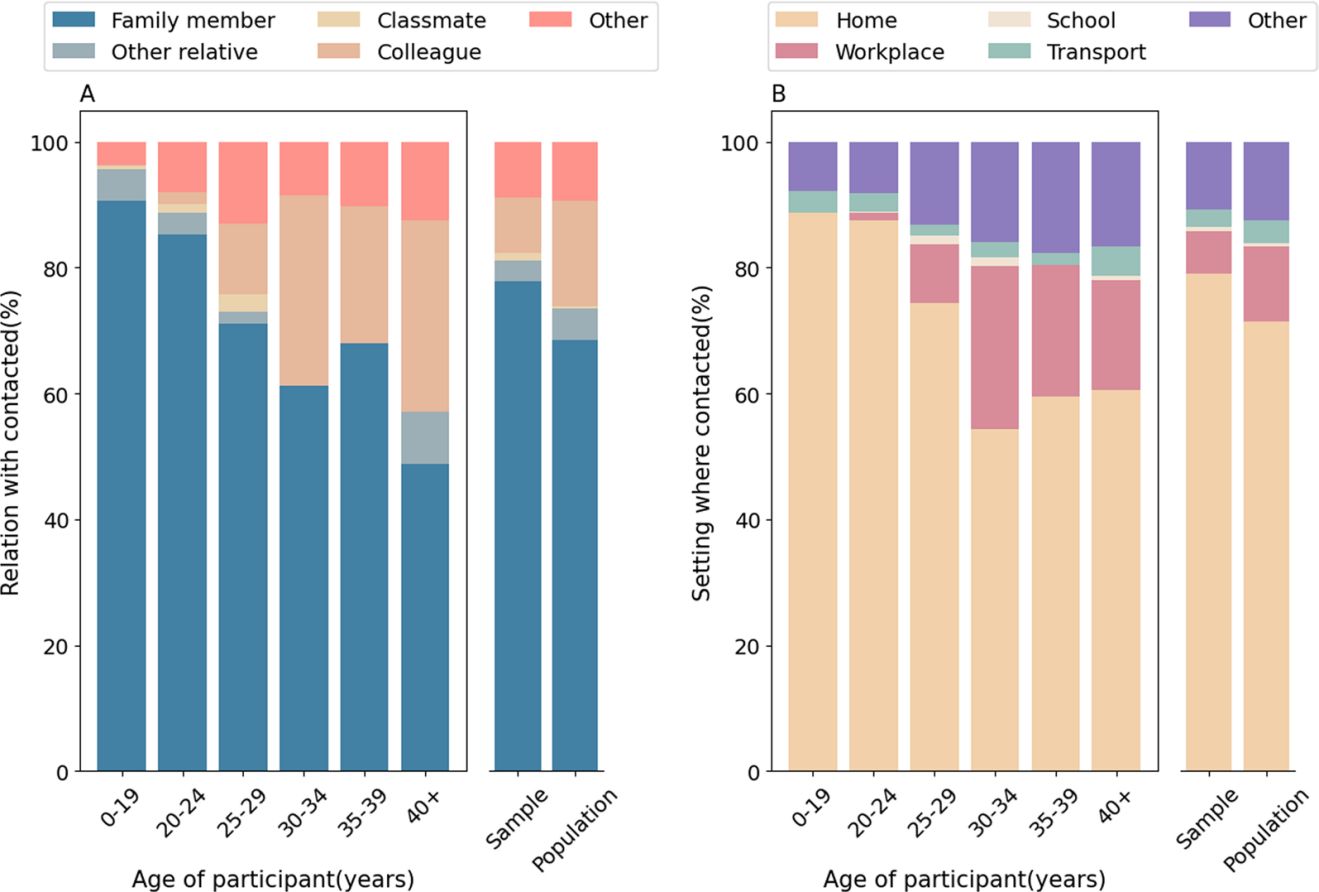
Relationship between contacts and location of contacts. **A** Probability distribution of the relationship between the study participant of a given age group and the contacted individual. The bars on the right show the probability distribution of the relation between study participant irrespective of age and the contacted individual in the sample and by adjusting for the age structure of the 12 provinces included in our analysis as reported in the Chinese 2010 census. **B** As **A**, but showing the location where contacts took place

As for the relationship between contacts, 77.9% of recorded contacts occurred between family members, 3.2% with other relatives, 1.2% classmates, 8.9% with colleagues, and 8.8% with other individuals (Fig. 2A). Despite the lockdown being lifted at the time of our survey, our results show that contacts were predominantly occurring within households. This trend is more marked for younger individuals: for individuals aged 0–19 years, 90.7% of contacts occurred with household members, while this figure decreases to 48.8% for individuals aged 40 years or more. Depending on the age group, the proportion of contacts with work colleagues varies in the range 0.3%-30.4% (Fig. 2A). The fraction of contacts with other relatives varied between 3.6% and 13.1%, with the larger fraction found in the age groups 0–19 and 40 + .

We estimated that the household was the most common place where contacts took place (79.1%), followed by “other places” (10.8%), workplace (6.8%), transport (2.8%), and school (0.6%) (Fig. 2B). Individuals aged 40 + years made about 17.4% of their contacts in the workplace.

### Contact duration

**Fig. 3:**
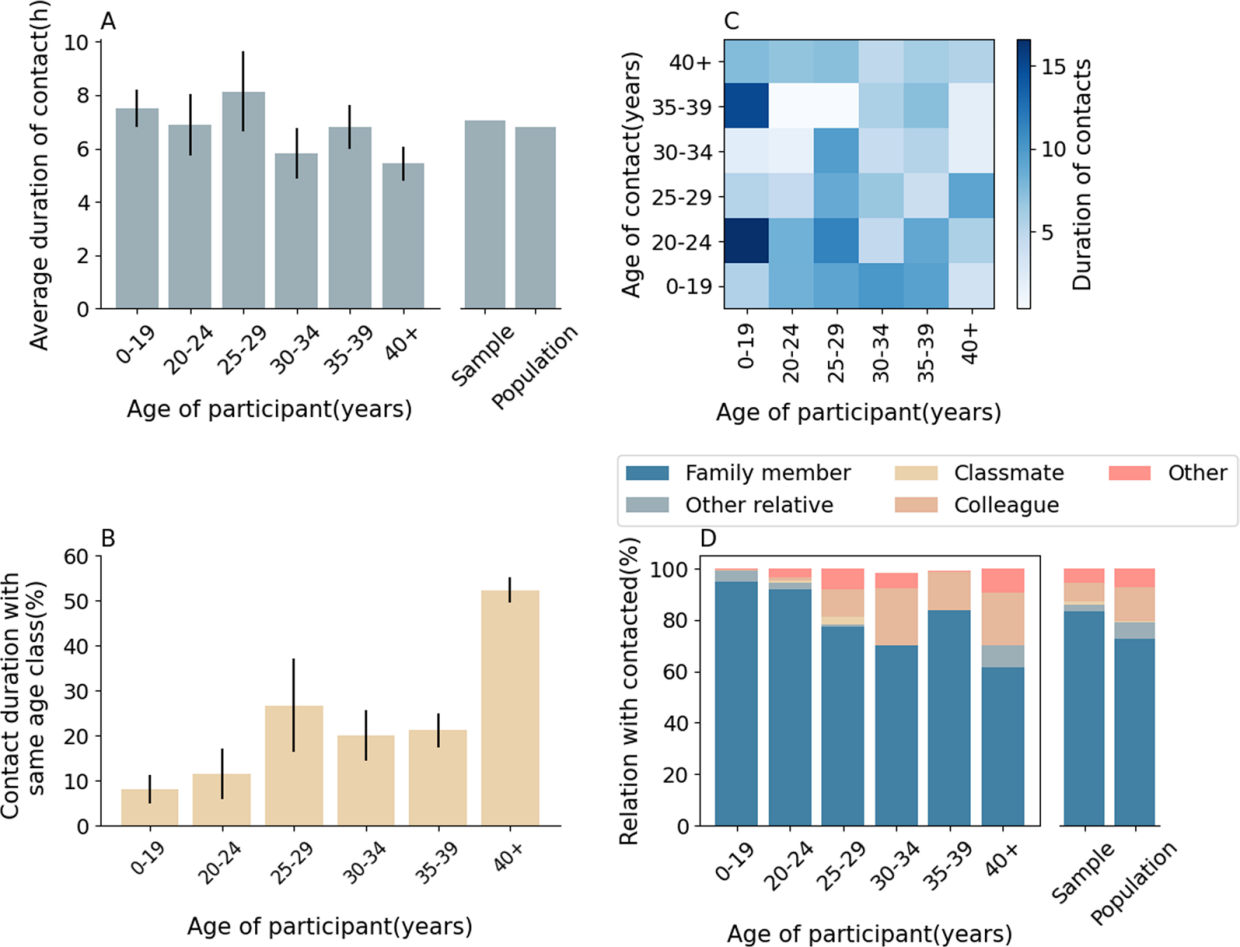
Daily average duration of contacts by age. **A** Daily average duration of contacts by age group of study participants. The standard error in each age group is obtained from bootstrap sampling according to the age distribution for the 12 provinces included in our analysis as reported in the Chinese 2010 census. The bars on the right show the daily average duration per contact between study participant irrespective of age and the contacted individual in the sample and by adjusting for the age structure of the 12 provinces included in our analysis. **B** Fraction of contact duration with same age class individuals. **C** Overall contact duration matrix by age groups. **D** Probability distribution of the relation between study participant of a given age group and contacted individuals

In this survey, the contact duration per contact was also collected. As shown in Fig. 3A, study participants presented an average contact duration per contact from 5.4 to 8.1 h. Individuals aged 40 + years showed the shortest average duration per contact, while participants in age group 25–29 show the longest average duration per contact. Similarly, no significant differences can be seen in the duration per contact after adjusting for the actual age distribution of the focus population (Fig. 3A). Participants over 40 had spent the most amount of time with same-age contacts, while individuals in age groups 0–19 and 20–24 spent the lowest amount of time with same-age contacts (Fig. 3B). The average durations per contact between individuals of different age groups are reported in Fig. 3C. As for the relationship between contacts, the duration that participants had contact with family members comprised the majority of the total time, mainly at 83.3% (Fig. 3D), followed by time spent with colleagues at 7.4%.

### Impact of contact patterns on COVID-19 burden

**Fig. 4:**
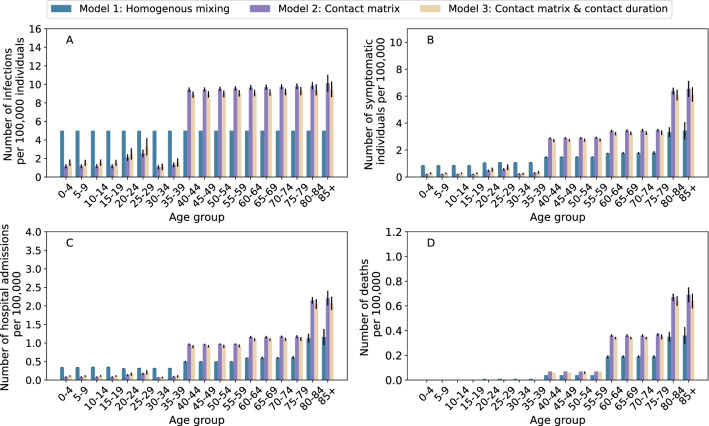
Application to COVID-19. **A** Number of infections by age group for the three models. The number of initial seeds is set to 1, ${\mathrm{R}}_{0}$ is fixed to 1.3, and the simulation is interrupted when cumulative 1,000 infections are reached. **B** As **A**, but for symptomatic individuals. **C** As A, but for hospital admissions. **D** As **A**, but for deaths

The simulation results show, for each indicator of the disease burden, marked differences between the homogeneous mixing model (Model 1) and the two models considering heterogeneous contacts by age (Model 2 and Model 3), see Fig. 4. On the other hand, including contact duration (Model 3) yields to results comparable to that of the model considering the number of contacts by age only (Model 2). These results are consistent when considering different numbers of initial seeds and different values of the reproduction number (Additional file 1: Figs. S2 and S3). The estimated age-distribution of COVID-19 burden is unaltered when different numbers of cumulative infections to interrupt simulation are considered, while the absolute burden is proportional to them (Additional file 1: Fig. S4).

## Discussion

In this paper, we analyzed contact intensity from two different angles: contact number and average contact duration. Overall, individuals reported an average of 2.3 contacts per day (IQR: 1.0–3.0) and 7.0 h per contact (IQR: 1.0–10.0). The estimated number of contacts well compares with estimates obtained in February 2020 for Wuhan (2.0 contacts on average) and Shanghai (2.3 contacts on average) [16]. Also the estimated contact duration is in line with previous estimates obtained in a 2015/16 survey conducted in Hong Kong where the authors estimated an average contact duration of 9.3 h [6], which falls inside our interquartile range. We estimated both the average number of contacts and contact duration to increase as the household size increases. A similar trend was estimated in Hong Kong before the COVID-19 pandemic [6].

As for the relationship between contacts, we estimated the large majority of contacts (77.9%) to occur between family members, highly different from pre-pandemic studies (range: 22.9–25.8%) [7, 27] but consistent with other studies in the early phase of the COVID-19 pandemic (range: 63.0–94.1%) [16, 28].

In the post-lockdown period, when workers had started to resume in-person work, non-negligible percentages of contact number and contact duration were recorded in the workplace with colleagues. However, contacts reported with family members at home still represented a remarkable fraction of overall recorded contacts and contact duration, indicating that during the study period people were still cautious about resuming contacts.

The following limitations need to be considered when interpreting the results of the survey presented in this study. The current survey over-samples school-age individuals and participants needed to be able to have access to the internet, potentially biasing our results. Recorded contacts can be affected by recall bias and compliance bias as well. A discussion about the biases commonly affecting contact survey studies can be found in Smieszek et al. [29]. Another limitation is that only the total time the participant spent in the same room/environment with a contacted individual was recorded; it is unknown whether this definition of contact duration is adequate for studying transmission patterns of respiratory pathogens. Most schools were still closed (online learning) during the study period; this does not allow us to draw conclusions about the post-lockdown mixing patterns of Chinese students. This study is based on limited number of participants (354) that does not allow us to consider refined age groups. In particular, we did not have enough sample to subdivide individuals aged 40 + years in multiple subgroups. As such, we are not able to draw specific conclusions for the contact patterns followed by the elderly—a key group in determining COVID-19 burden. Finally, the modeling exercise presented here does not have the ambition of being representative of the all complexities of COVID-19 epidemiology; it serves as a simplified example to highlight the effect of mixing patterns in SARS-CoV-2 transmission patterns and COVID-19 burden.

Our modeling exercise highlights the effect of considering human mixing patterns in shaping SARS-CoV-2 transmission patterns and COVID-19 burden. Considering age-mixing patterns provides remarkably different estimates of COVID-19 burden by age group as compared to a model where the population mixes fully at random. Considering contact duration in addition to the number of contacts by age lead to comparable results to that of the model accounting for the number of contacts only. The small sample size of our survey does not allow a fine characterization of contact patterns of individuals aged 40 + years. A more refined resolution in this age segment of the population is warranted to provide deeper insights on SARS-CoV-2 transmission patterns and COVID-19 burden.

This work can be considered as an incremental step in quantifying contact patterns by age, relationship, social setting, and duration in China during the COVID-19 pandemic.

## Supplementary Information

**Additional file 1.** Model parameters and sensitivity analysis results.

## Data Availability

The datasets generated and/or analyzed in the current study are available in the [Sichuan_Contact_Survey] repository, [https://github.com/QH-Liu/Sichuan_Contact_Survey].
