# Supplementary material for: Quantifying human mixing patterns in Chinese provinces outside Hubei after the 2020 lockdown was lifted
Source: BMC Infect Dis. 2022 May 21;22:483. doi: 10.1186/s12879-022-07455-7 (PMC9123295; doi:10.1186/s12879-022-07455-7)
Supplement: Supplementary file 1 — Additional file 1. Model parameters and sensitivity analysis results. [file 12879_2022_7455_MOESM1_ESM.docx]

**Appendix A**

**Table S1. Parameters regulating COVID-19 burden.**

| **Description** | **Age (years)** | **Value (%)** | | **Reference** |
| --- | --- | --- | --- | --- |
| Probability of developing respiratory symptoms and/or fever given infection | 0-19  20-39  40-59  60-79  80+ | 18.1  22.4  30.5  35.5  64.6 | | Poletti et al. [1] |
| Proportion of laboratory-confirmed symptomatic individuals requiring hospitalization | 0-19  20-39  40-59  60+ | 40.0  29.2  33.3  33.8 | | Yang et al. [2] |
| Fatality ratio among laboratory-confirmed symptomatic individuals | 0-19  20-39  40-59  60+ | | 0.51  0.65  2.38  10.52 | Yang et al. [2] |

**Table S2. Transmission Parameters for all simulations.**

| **Scenario** | **Parameters** | **Model 1** | **Model 2** | **Model 3** |
| --- | --- | --- | --- | --- |
| Baseline | $R_{0}$ | 1.3 | 1.3 | 1.3 |
|  | $\beta$ | 0.0052775 | 0.0030013 | 0.0038085 |
|  | $\gamma$ | 1/5.1 | 1/5.1 | 1/5.1 |
|  | Initial seeds | 1 | 1 | 1 |
|  | Cumulative infections reached to interrupt simulation | 1,000 | 1,000 | 1,000 |
|  | Population size | 758,529,559 | 758,529,559 | 758,529,559 |
| Sensitivity analysis on the initial number of seeds | Initial seeds | - | - | 5, 20 |
| Sensitivity analysis on the reproduction number | $R_{0}$ | - | - | 2.0 |
|  | $\beta$ | - | - | 0.0058425 |
| Sensitivity analysis on the cumulative number of infections reached to interrupt the simulation | Cumulative infections reached to interrupt simulation | - | - | 500, 2,000 |

Parameters that are not listed in sensitivity analyses are kept as in the baseline analysis.

**Table S3. Data completeness.**

| **Province** | **Total Number of**  **Participants** | **Number of Participants with Missing Information** | **Number of Participants with Complete Information** |
| --- | --- | --- | --- |
| Sichuan | 360 | 160 | 200 |
| Chongqing | 34 | 14 | 20 |
| Shandong | 33 | 14 | 19 |
| Hebei | 23 | 6 | 17 |
| Henan | 25 | 9 | 16 |
| Zhejiang | 21 | 7 | 14 |
| Fujian | 18 | 5 | 13 |
| Yunnan | 23 | 10 | 13 |
| Hunan | 19 | 7 | 12 |
| Guangdong | 19 | 9 | 10 |
| Jiangxi | 17 | 7 | 10 |
| Shanxi | 21 | 11 | 10 |
| Total | 613 | 259 | 354 |

One participant was not a Chinese resident and one responded to the survey outside of the 20-day study period; as such, they were excluded from the study.

**Figure S1. Age distribution.**


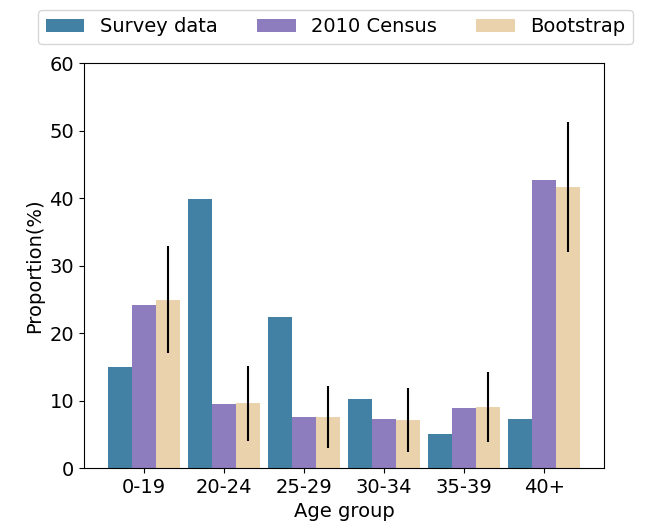


**Figure S1**: Age distributions of study participants, the 12 analyzed provinces from the 2010 Chinese census (2010 census [3]) and the bootstrap sample. Note that standard errors are given for the bootstrapped data.

**Figure S2. Sensitivity analysis on the initial number of seeds**


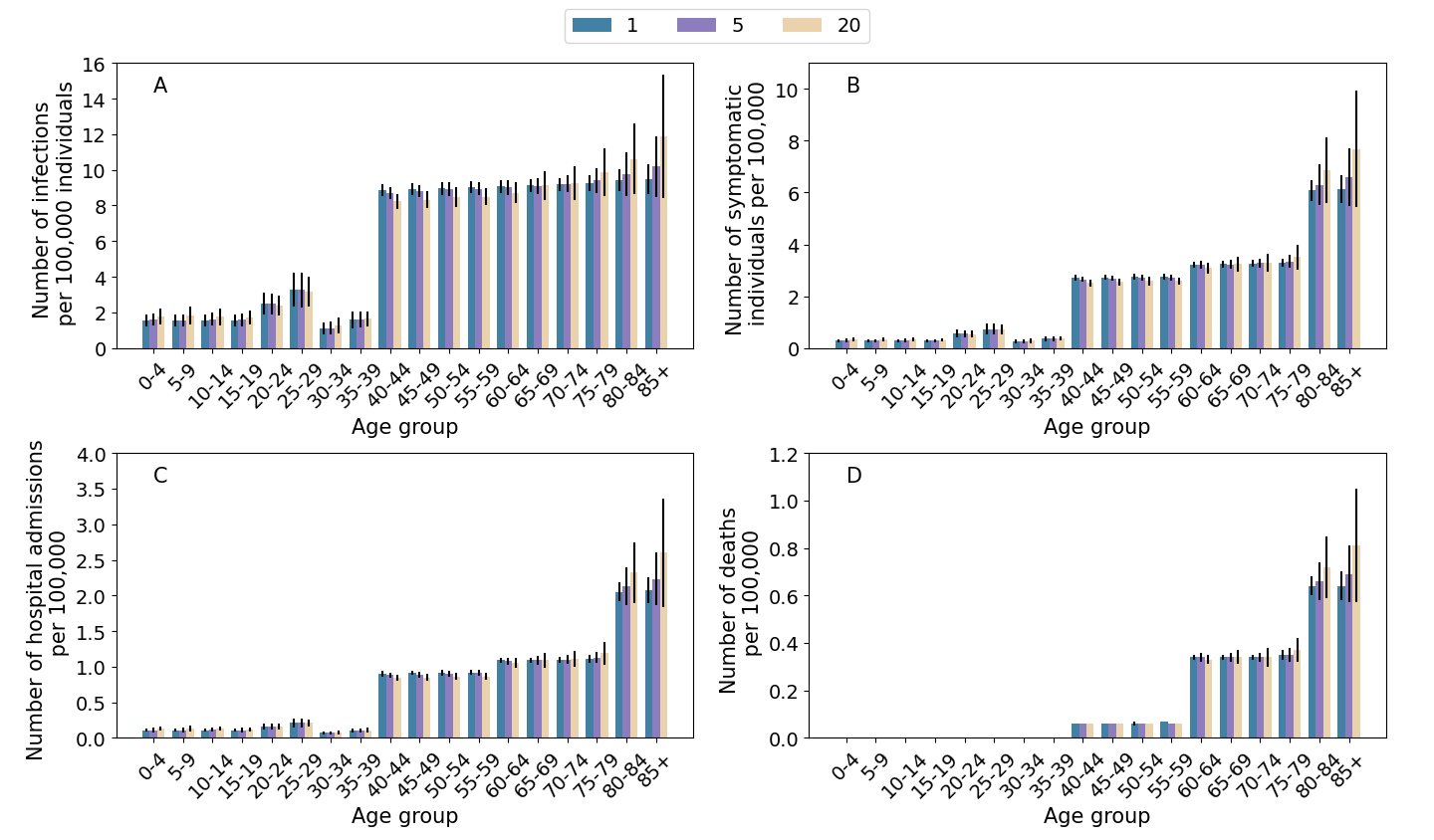


**Figure S2**: Sensitivity analysis on the initial number of seeds for Model 3**. A** Number of infections by age group for Model 3 when varying the number of initial seeds; $R_{0}$ is fixed to 1.3 and the simulation is interrupted when cumulative 1,000 infections are reached. **B** As A, but for symptomatic individuals. **C** As A, but for hospital admissions. **D** As A, but for deaths.

**Figure S3. Sensitivity analysis on the reproduction number**


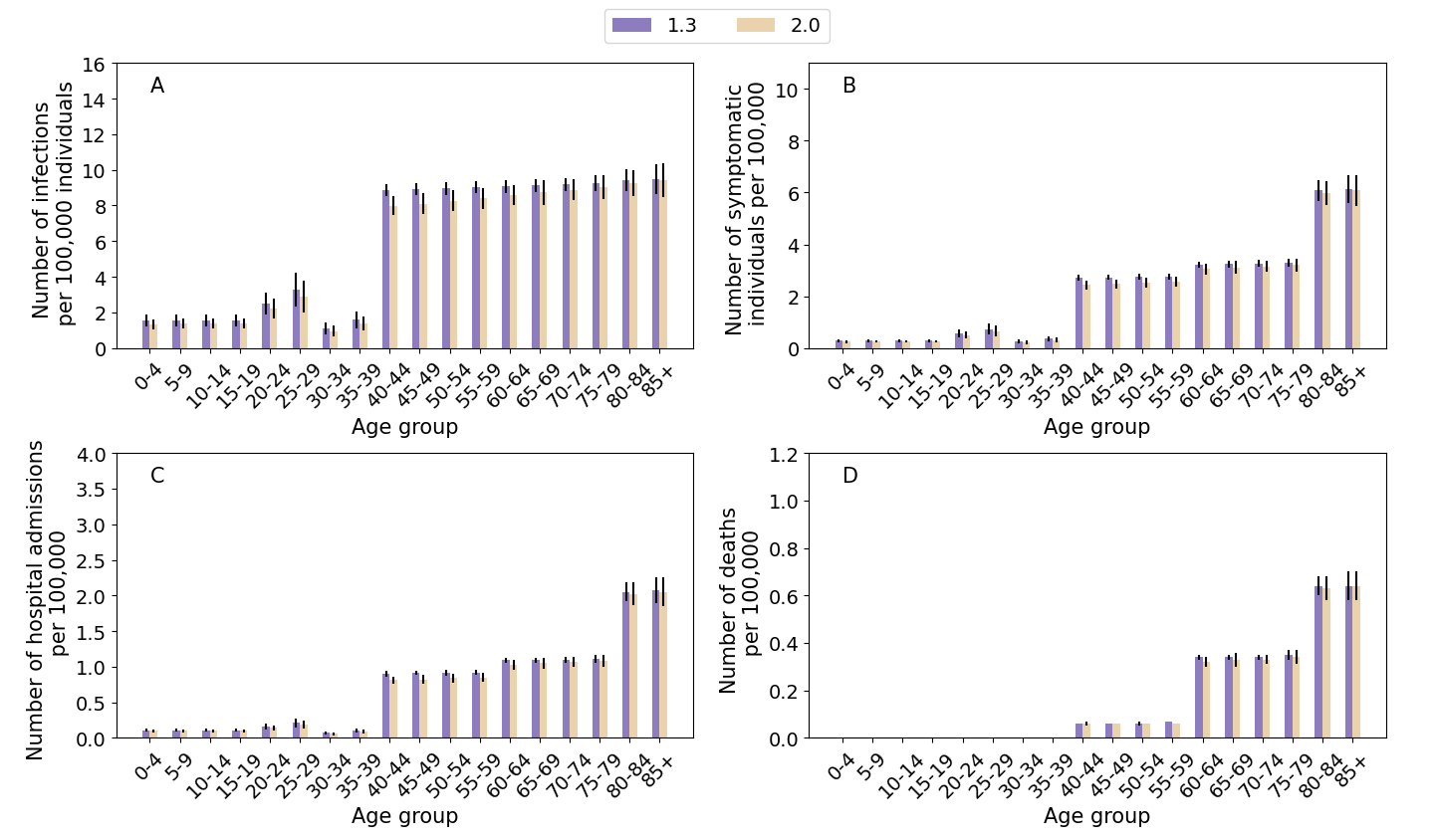


**Figure S3**: Sensitivity analysis on the reproduction number for Model 3. **A** Number of infections by age group for Model 3 when varying the reproduction number. The initial number of seeds is set to 1 and the simulation is interrupted when cumulative 1,000 infections are reached. **B** As A, but for symptomatic individuals. **C** As A, but for hospital admissions. **D** As A, but for deaths.

**Figure S4. Sensitivity analysis on the cumulative number of infections reached to interrupt the simulation**


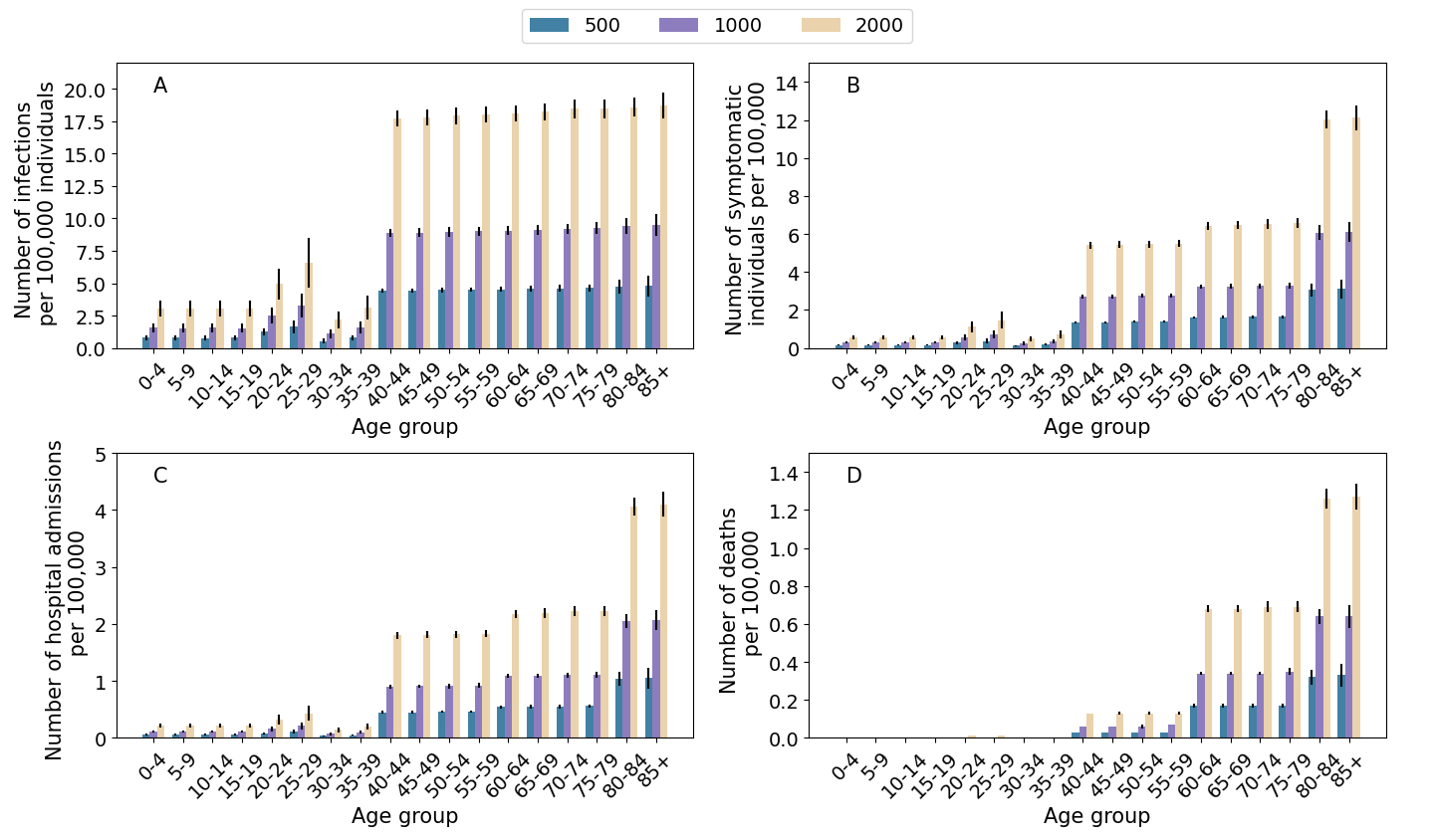


**Figure S4**: Sensitivity analysis on the cumulative number of infections reached to interrupt simulation for Model 3. **A** Number of infections by age group for Model 3 when varying the cumulative number of infections reached to interrupt the simulation; $R_{0}$ is fixed to 1.3 and the initial number of seeds is set to 1. **B** As A, but for symptomatic individuals. **C** As A, but for hospital admissions. **D** As A, but for deaths.

**References**

1. Poletti P, Tirani M, Cereda D, et al. Association of age with likelihood of developing symptoms and critical disease among close contacts exposed to patients with confirmed sars-cov-2 infection in italy. JAMA Network Open, 2021, 4(3): e211085-e211085.
2. Yang J, Chen X, Deng X, et al. Disease burden and clinical severity of the first pandemic wave of COVID-19 in Wuhan, China. Nature Communications, 2020, 11(1): 1-10.
3. National Bureau of Statistics of the People's Republic of China. China Statistical Yearbook 2010. Available in Chinese at http://www.stats.gov.cn/tjsj/ndsj/2010/indexch.htm. (Accessed on Nov. 25, 2021).
